# Chitosan improves the durability of resin-dentin interface with etch-and-rinse or self-etch adhesive systems

**DOI:** 10.1590/1678-7757-2021-0356

**Published:** 2021-12-13

**Authors:** Vitória Leite PASCHOINI, Isabella Rodrigues ZIOTTI, Cláudio Roberto NERI, Silmara Aparecida Milori CORONA, Aline Evangelista SOUZA-GABRIEL

**Affiliations:** 1 Universidade de São Paulo Faculdade de Odontologia de Ribeirão Preto Departamento de Odontologia Restauradora Ribeirão Preto Brasil Universidade de São Paulo, Faculdade de Odontologia de Ribeirão Preto, Departamento de Odontologia Restauradora, Ribeirão Preto, Brasil.; 2 Universidade de São Paulo Faculdade de Filosofia, Ciências e Letras Departamento de Química Ribeirão Preto Brasil Universidade de São Paulo, Faculdade de Filosofia, Ciências e Letras, Departamento de Química, Ribeirão Preto, Brasil.

**Keywords:** Adhesive, Bond strength, Chitosan, Dentin, FTIR

## Abstract

Degradation of the dentin-resin interface can occur due to hydrolysis of exposed collagen, resulting in reduced bond strength. This study assessed the effect of dentin treatment with chitosan combined with an etch-and-rinse or self-etch adhesive system on improvement of bond strength and preservation of the interface durability.

**Methodology:**

Enamel was removed from 80 molars and the teeth were divided into two groups: without chitosan (control) or with 2.5% chitosan gel (1 min). They were further subdivided into two subgroups according to the adhesive system: etch-and-rinse or self-etch. Dentin was restored using a composite resin. Half of the specimens from each restored group were subjected to interface aging and the remaining specimens were used for immediate analysis. The specimens were sectioned and subjected to microtensile bond strength (µTBS) test (n=10), chemical composition testing using Fourier-transform infrared (FTIR) spectroscopy (n=4) and energy-dispersive spectroscopy (EDS) (n=5), and morphological analysis of the adhesive interface using scanning electron microscopy (SEM) (n=5). Data were analyzed using three-way ANOVA.

**Results:**

Chitosan improved the µTBS of the adhesive interface when compared with the control group (p=0.004). No significant differences were observed in dentin adhesion between the adhesive systems (p=0.652). Immediate µTBS was not significantly different from that after 6 months (p=0.274). EDS and SEM did not show significant differences in the chemical and structural composition of the specimens. FTIR showed a decrease in the intensity of phosphate and carbonate bands after using chitosan.

**Conclusions:**

Dentin treatment with chitosan combined with an etch-and-rinse or self-etch adhesive system improved the immediate and preserved the 6-month bond strength of the adhesive interface.

## Introduction

In dental procedures, adhesives are combined with composite resin to create a strong bond to resolve many restorative issues.^1^ Different adhesive protocols that can achieve a hybrid layer include the etch-and-rinse and the self-etch strategies.^2^

An etch-and-rinse adhesive system applies phosphoric acid at a concentration between 30% and 40%^3^to remove smear layer. Dentin is demineralized up to a depth of 3–5 µm, exposing the collagen fibrils and allowing adhesive and resin infiltration.^6^ Therefore, mechanical interlocking of resin tags within the acid-etched surface provides a favorable bond to the dental substrate.^4,6^ However, since dentin adhesion is more challenging than enamel adhesion, self-etch adhesives were introduced to control the sensitivity of the etch-and-rinse technique to humidity and also to simplify the procedure.^7,8^ Moreover, acid etching of dentin can damage the collagen matrix and decrease the durability of the restorative treatment.^5^

A self-etch system incorporates the smear layer into the hybrid layer through acid monomers, exposing type I collagen fibrils from the dentin. Simultaneously, the resin monomers penetrate the smear layer into mineralized dentin.^7^ Although etch-and-rinse adhesives are still the gold standard for dental adhesion, studies have reported promising bond strengths with dentin using self-etch systems.^7^

The dentin collagen matrix is composed of endogenous metalloproteinases, enzymes that can accelerate collagen degradation. Enzymes such as matrix metalloproteinase (MMP)-2, MMP-9 gelatinases, and MMP-8 collagenase could be induced by insufficient adhesive infiltration into the exposed collagen fibrils, reducing the formation of the hybrid layer.^1,9^ This process interferes negatively with the bond strength of the restorative material to dentin.^10^ Therefore, substances that prevent such problems are being applied to the dentin surface, allowing greater stability via cross-linking between the collagen fibrils and the organic matrix.^1,2^

Chitosan biopolymer has been highlighted among substances that can decrease degradation of the collagen matrix caused by metalloproteinases.^2^ Chitosan is derived from chitin, a copolymer obtained from crustacean carapace, fungi, and insects.^11^ It has a high molecular weight and is composed of B-2-amino-2-deoxy-D-glucose (or D-glucosamine) derived from deacetylation of chitin.^12,13^ Its structure stands out in terms of reactivity, since it contains the amino group, which allows substitution reactions.^13^ Different from another MMP inhibitors, chitosan is a promising active material due to favorable features combined in its composition, such as high biocompatibility,^11^ durable hygroscopic nature,^14^ chelating capacity,^15^ antimicrobial property,^14^ and bioadhesive interaction with dental tissues.^13,15^ Chitosan can form cross-links with dentin collagen, strengthening the fibrils against degradation, besides decreasing the action of MMPs.^2^ In dentistry, chitosan is widely applied in periodontitis, bone tissue repair, endodontics, enamel remineralization,^16^ and particularly in restorative dentistry to improve the adhesive infiltration and to increase the bond strength of resin to dentin.^12,17^

To the best our knowledge, few studies have investigated better dentin-resin bonds^2,18^ using adhesive protocols with chitosan. The null hypotheses of the study were: 1) Chitosan gel combined with an etch-and-rinse or self-etch adhesive system would exhibit no significant difference in microtensile bond strength of resin to dentin (µTBS); 2) There is no difference in the amount of the chemical elements of dentin with or without the chitosan gel, using EDS; 3) Morphology of the adhesive interface would not exhibit difference among groups; 4) There is no difference in the bands intensity of the chemical substances of dentin after using chitosan combined with etch-and-rinse or self-etch adhesive system, using FTIR.

## Methodology

### Estimation of sample size

A pilot study (n=3) was conducted for the µTBS test to estimate the number of dental specimens required to find differences between the control group and at least one experimental group. Similarly, literature was consulted to estimate the effect size.^17^ Power analysis was performed using the G*Power software (alpha=0.05 and power=0.85) and a minimum sample size of 10 specimens was deemed adequate.

### Experimental design

**Figure 1:**
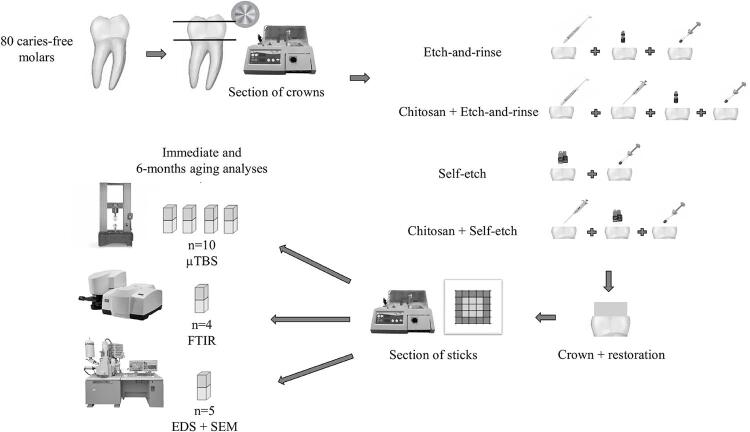
Schematic illustration of the experimental design

The sample consisted of 80 caries-free molars (10 teeth for each subgroup). The factors intended to be analyzed were 1) dentin treatment: without chitosan (control) or with 2.5% chitosan gel, 2) the adhesive system: etch-and-rinse or self-etch, and 3) aging of the adhesive interface: no aging (tests after 24 h) or aging (tests after 6 months of water storage + enzymatic degradation). The response variables were: 1) µTBS of the resin-dentin interface and the modes of failure (n=10), 2) chemical composition of the adhesive interface analyzed using Fourier-transform infrared (FTIR) (n=4) spectroscopy and energy-dispersive spectroscopy (EDS) (n=5), and 3) morphology of the adhesive interface analyzed using scanning electron microscopy (SEM) (n=5). Figure 1 shows the schematic representation of the experimental design.

### Sample selection and preparation

This study was approved by the local research ethics committee (protocol: #90731618.2.0000.5419). In total, 80 sound human molars, recently extracted, were selected from the local Biobank. The teeth were immersed in 0.1% thymol solution at 4°C until use^9^ and were subsequently washed in running water for 24 h to eliminate the residues of the solution. The teeth were then analyzed with a stereoscopic magnifying glass (Nikon, Melville, NY, USA) to verify the absence of structural defects.

Occlusal enamel of the molars was removed with a diamond-coated disc attached to a precision cutting machine (lsomet 1000; Buehler, Lake Bluff, IL, USA) to expose the dentin surface. The roots were sectioned horizontally 1 mm below the cementoenamel junction. The dentin surface was polished with a 600-grit silicon carbide sandpaper (Hermes Abrasives Ltda, Virginia Beach, VA, USA) for 30 s in a water-cooled polishing machine (Arotec, Cotia, SP, Brazil) and analyzed under a stereoscopic microscope (40×) (Leica Microsystems, Wetzlar, Germany) to ensure that all the enamel was removed.

### Preparation of the chitosan gel

The experimental chitosan gel (2.5%) was prepared according to the method described in previous studies by our research group,^17,19^ in which the results were favorable for this gel concentration. A commercially available (Sigma Aldrich, Saint Louis, MO, USA) low molecular weight (75–85% deacetylation) chitosan was used.^20^ Two and a half grams of chitosan were added slowly to 100 mL of 1% acetic acid solution under constant magnetic stirring (Marconi, Piracicaba, SP, Brazil) for 20 min (time required to solubilize the polysaccharide, and for the mixture to obtain a gel consistency). To avoid the aggregation of particles and to neutralize the pH, 1 mol/L of NaOH was added to the gel, achieving the pH=6.2.

### Experimental groups

In total, 40 dentin specimens treated with 2.5% chitosan gel and 40 specimens without chitosan (control) were randomly subdivided into two groups according to the adhesive system: etch-and-rinse or self-etch adhesive. Each group was again divided into two subgroups (10 teeth for each subgroup) according to the aging protocol of the adhesive interface: no aging or aging (6 months of water storage + enzymatic degradation).

### Restorative procedure

The protocols of each experimental group were:

Etch-and-rinse adhesive: the surface was conditioned with 37% phosphoric acid (Condac, FGM, São Paulo, Brazil) for 15 s and then washed with distilled water for a similar duration. The adhesive system was applied according to the manufacturers’ instructions.

Chitosan application followed by etch-and-rinse adhesive: the surface was conditioned with 37% phosphoric acid (Condac, FGM, São Paulo, Brazil) for 15 s and then washed with distilled water for a similar duration, then dentin was irrigated with 0.5 ml chitosan gel, which remained on the surface for 1 min, followed by washing for 15 seconds and drying with absorbent paper.^19^ The adhesive system was applied according to the manufacturers’ instructions.

Self-etch adhesive: the adhesive system was applied according to the manufacturers’ instructions.

Chitosan application followed by self-etch adhesive: the dentin was irrigated with 0.5 ml chitosan gel, which remained on the surface for 1 min, followed by washing for 15 seconds and drying with absorbent paper.^19^ The adhesive system was applied according to the manufacturers’ instructions.

For the etch-and-rinse adhesive system, Adper Single Bond 2 (3M ESPE, St. Paul, MN, USA), two layers of the adhesive were applied actively using a microbrush applicator, with subsequent solvent evaporation and polymerization using a light-emitting diode (LED) source (Gnatus, Ribeirão Preto, SP, Brazil) for 20 s. For the self-etch adhesive system, Clearfil SE Bond (Kuraray, Kurashiki, Okayama, Japan), one layer of primer was actively applied with a microbrush applicator for 20 s, followed by volatile compound evaporation with a mild air stream. Subsequently, one layer of bond was applied and polymerization was performed using the same LED source for 10 s.

The dentin surfaces were restored using composite resin (Filtek Z250, 3M, ESPE, St. Paul, MN, USA). The resin was applied in two increments of 2 mm each and polymerized using the LED light source (Gnatus, Ribeirão Preto, SP, Brazil) for 20 s, maintaining the device tip at a distance of 1 cm from the resin surface. The maximum polymerization power of the LED source was 1200 mW/cm^2^ and the wavelength was between 420 nm and 480 nm, which was monitored using a radiometer (RD7; Ecel Indústria e Comércio Ltda, Ribeirão Preto, SP, Brazil).

### Sectioning the specimens

Half of the specimens from each restored group (etch-and-rinse or self-etch) were subjected to immediate analysis. They were stored in distilled water at 37°C for 24 h and then sectioned in stick forms with a cross-sectional area of 1.0±0.2 mm^2^ using a precision cutter under constant irrigation. The sticks were removed from the central portion of the specimen, avoiding pre-testing failures. The thickness of the sticks was confirmed using a digital caliper (Mitutoyo, Tokyo, Japan). Ten sticks were used for the adhesive strength test, four were used for FTIR spectroscopy analysis, and five slices from the margin of the restoration were used for EDS and SEM analyses.

### Aging process

The specimens intended to undergo aged interface analysis underwent aging before the sectioning process. This process involved a combination of hydrolytic^21,22^and enzymatic degradation.^23^ For hydrolytic aging of the interface, specimens were stored in 20 mL distilled water at 37°C for 6 months with weekly water exchange.^22^ Subsequently, the specimens were subjected to enzymatic degradation by immersing in artificial saliva with 100 U/mL of *Clostridium histolyticum* collagenase (Sigma-Aldrich, Saint Louis, MO, USA) for 5 days at 37°C,^24^ followed by washing with distilled water, drying, and sectioning in the same manner as described for the immediate analysis.

### Analysis of µTBS

The specimens were fixed in a stainless-steel device using cyanoacrylate adhesive (Super Bonder, Henkel Ltda, São Paulo, SP, Brazil) and placed in a universal testing machine (Instron Corporation, Canton, MA, USA) under a force of 50 kg, at a cross head speed of 0.5 mm/min. The adhesive strength values were expressed in megapascals (MPa) using the cross-sectional area of the sticks measured before the test. The specimen surfaces were analyzed using a stereoscopic microscope (40×) (Leica Microsystems, Wetzlar, Germany) to categorize the fracture patterns. The fractures were classified as adhesive fractures, when a thin layer of adhesive material covered the dentin surface; cohesive fractures of the material, when the surface was covered by composite resin; cohesive fractures of the substrate, when the failure occurred in dentin; and mixed fractures, when a combination of adhesive and cohesive fractures was observed.^3^

### EDS and SEM analyses of the adhesive interface

EDS analysis is based on emission of energy from electron beam in the sample to identify and quantify the chemical elements present on it.^25^ The slices intended to undergo EDS and SEM analyses were fixed in acrylic resin and their interface was polished with decreasing grits of sandpaper (#600 and #1200) and a wet synthetic fiber polishing cloth (Buehler, São Paulo, SP, Brazil) with alumina slurry of 0.3 μm granulation (Buehler, São Paulo, SP, Brazil). Subsequently, the specimens were washed, dried, and fixed in stubs with double-sided carbon tape and placed under a scanning electron microscope with an EDS-coupled detector (EVO 50; Carl Zeiss, Cambridge, England). The entire adhesive interface was displayed under a magnification of 100× to determine the percentages of the predominant chemical elements (%).

Subsequently, the specimens were dehydrated in 25, 50, 75, and 95 °GL ethanol by immersion for 20 min in each solution and then immersed in 100 °GL ethanol for 1 h. They were fixed again in metallic stubs and covered with a thin layer of gold-palladium alloy in a vacuum metallization apparatus (Bal-Tec SCD 005 Sputter Coater, Balzers, Liechtenstein). Adhesive interface was completely scanned and a more representative area of each group was photographed at different magnifications. The presence and uniformity of the hybrid layer and the tags in the adhesive interface were observed.

### FTIR spectroscopy analysis

The FTIR spectroscopy analysis includes information regarding the chemical composition of the adhesive interface.^20^ Our study considered the organic and inorganic constituents in a qualitative analysis of the dentin adhesive interface. The sticks intended to undergo this analysis were placed on an attenuated total reflectance detector (ATR) coupled to a Fourier-transform spectrometer (IR Prestige-21, Shimadzu, Tokyo, Japan). The ATR allows analysis of solid samples with a plan and polished surface. The specimen was positioned on the press device so that light could exactly achieve the adhesive interface and provide the adequate spectra.

The spectra were acquired with a resolution of 2.0 cm^-1^ in the spectral region of 600–4000 cm^-1^ including 15 scans suitable for the acquisition of each spectrum. The transmittance was analyzed using Origin 8.0 (OriginLab, Northampton, MA, USA).

### Data analysis

The µTBS data were analyzed using IBM SPSS Statistics version 25 for Windows (IBM Corporation, Armonk, NY, USA) with a 5% significance level. Shapiro-Wilk test and Levene’s test ascertained a normal and homogeneous distribution of samples. Three-way analysis of variance was performed, considering adhesive (etch-and-rinse or self-etch), dentin treatment (with or without chitosan), and aging (immediate analysis or after 6 months) as independent factors. The bond strength was analyzed using the tooth as a statistical unit. The mean bond strength obtained from ten sticks of each tooth was used to represent the µTBS of that tooth, yielding 100 values per subgroup for the analysis.

FTIR data were explored in a qualitative chemical comparison at the adhesive interface among the experimental groups through transmittance of organic and inorganic compounds. The concentration of inorganic chemical elements was assessed using the spectral dispersive X-ray energy. SEM analysis of the photomicrographs was performed by two calibrated examiners (kappa>0.8).

## Results

### Analysis of µTBS

Table 1 presents the immediate and 6-month mean µTBS values of the specimens from different experimental groups. No statistically significant difference was observed (p=0.652) in dentin adhesion between the adhesive systems (etch-and-rinse and self-etch). Dentin treatment with chitosan significantly improved the bond strength (p=0.004) when compared with the control group (without chitosan). Immediate µTBS values were not significantly different from the 6-month µTBS values (p=0.274).

**Table 1:** The µTBS mean values (MPa) and standard deviations of the experimental groups

Adhesive	Baseline analysis (24 h)		After aging (6-months degradation)	
	without chitosan	with chitosan	without chitosan	with chitosan
Etch-and-rinse	31.48 ± 9.67^Ab^	37.25 ± 11.33^Aa^	28.94 ± 5.57^Ab^	38.00 ± 8.96^Aa^
Self-etch	28.02 ± 7.17^Ab^	33.89 ± 10.94^Aa^	31.44 ± 5.57^Ab^	36.77 ± 8.04^Aa^

Same capital letters denote groups that are not statistically different in the comparison within lines (p<0.05).

Same lowercase letters denote groups that are not statistically different in the comparison within columns (p<0.05).

**Figure 2:**
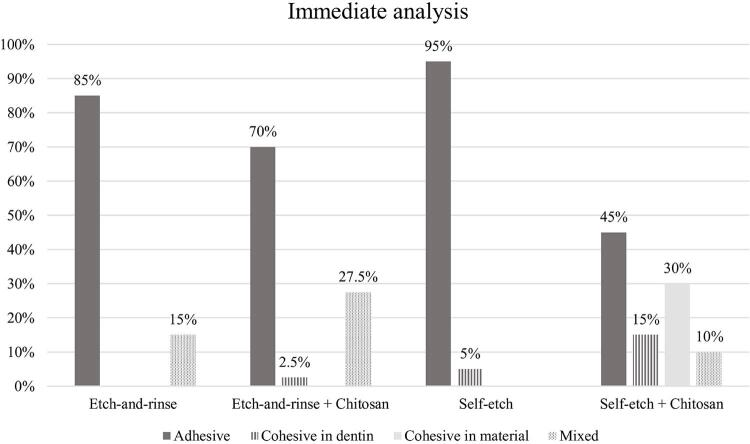
Failure pattern distribution (%) for microtensile bond strength of specimens (immediate analysis – 24 h)

**Figure 3:**
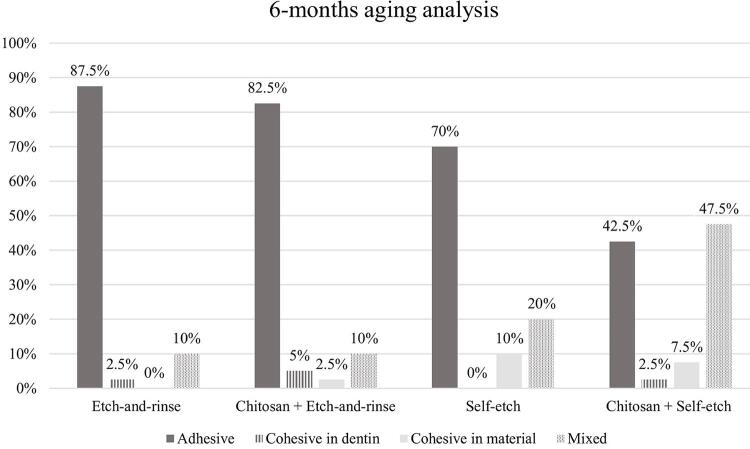
Failure pattern distribution (%) for microtensile bond strength of specimens after the aging process of the adhesive interface (6-months water-storage + bacterial degradation)

No significant interaction was observed between adhesive system and dentin treatment (p=0.975), between adhesive system and aging (p=0.515), between dentin treatment and aging (p=0.552), and among adhesive system, dentin treatment, and aging (p=0.535). Adhesive failure was predominant, except in the group treated with chitosan and restored with the self-etch adhesive system, which exhibited a greater number of mixed failures (Figure 2 and Figure 3).

### EDS and SEM analyses of the interface

EDS quantified carbon (C), oxygen (O), phosphorus (P), and calcium (Ca). Table 2 presents the data obtained from EDS analysis of the non-aged and aged specimens. The concentrations of C, O, P, and Ca remained stable in all groups. After the aging process, no significant difference was observed in these concentrations when compared with non-aged specimens.

**Table 2:** Mean and standard deviation of the atomic percentage (wt%) of specimens' elements

CE*	Baseline analysis (24 h)				After aging (6-months degradation)			
	without chitosan		with chitosan		without chitosan		with chitosan	
	Etch-and-rinse	Self-etch	Etch-and-rinse	Self-etch	Etch-and-rinse	Self-etch	Etch-and-rinse	Self-etch
C	(44.93 ± 6.9)	(37.98 ± 2.8)	(40.97 ± 2.9)	(39.73 ± 3.4)	(38.37 ± 0.4)	(38.68 ± 2.1)	(36.91 ± 2.2)	(39.57 ± 0.6)
O	(34.47 ± 2.0)	(42.98 ± 10.2)	(42.17 ± 2.7)	(37.52 ± 3.0)	(44.89 ± 3.6)	(41.97 ± 4.8)	(40.80 ± 4.7)	(40.20 ± 6.1)
P	(6.34 ± 1.4)	(5.73 ± 1.9)	(5.09 ± 0.0)	(6.80 ± 0.3)	(5.08 ± 0.8)	(5.96 ± 0.9)	(6.84 ± 0.8)	(6.17 ± 1.6)
Ca	(13.91 ± 3.3)	(12.85 ± 5.4)	(11.36 ± 0.1)	(15.65 ± 0.0)	(11.50 ± 2.2)	(12.96 ± 1.9)	(15.00 ± 1.7)	(13.53 ± 3.9)

*CE = chemical elements.

**Figure 4:**
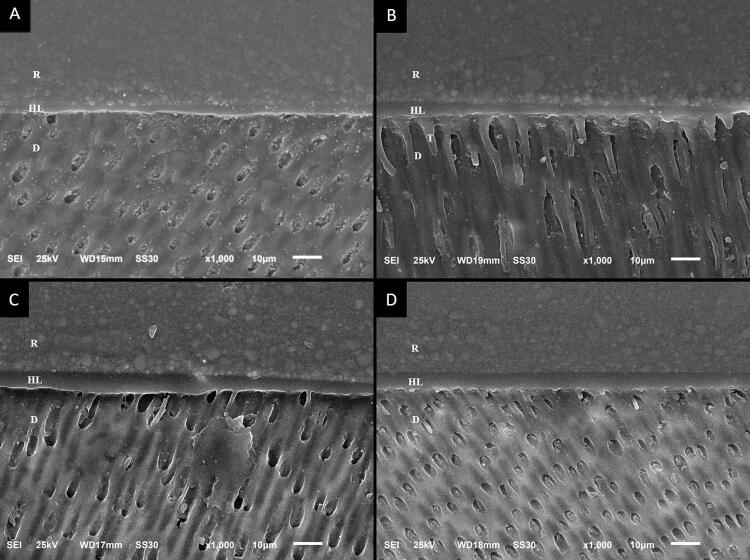
Photomicrographs (1000×) of the adhesive interface immediately after the adhesive procedure: (A) Etch-and-rinse adhesive; (B) Chitosan + Etch-and-rinse adhesive; (C) Self-etch adhesive (D) Chitosan + Self-etch adhesive. R, resin. HL, hybrid layer. D, dentin. T, resin tags

**Figure 5:**
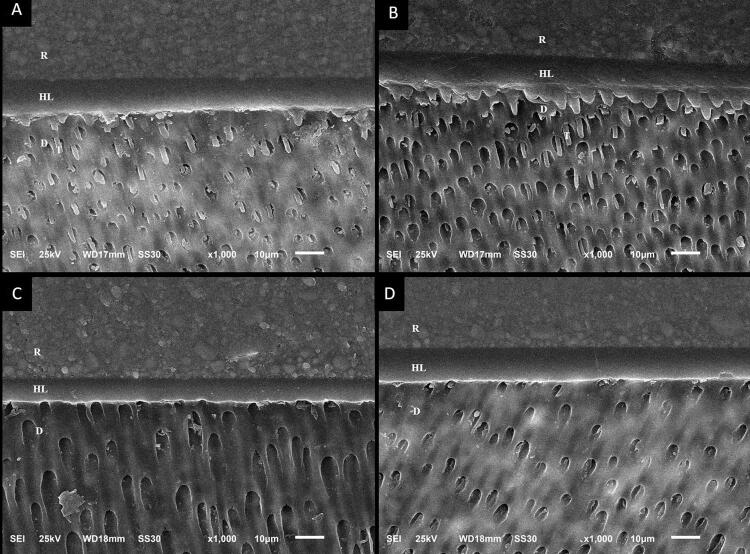
Photomicrographs (1000×) of the adhesive interface after 6-months aging: (A) Etch-and-rinse adhesive; (B) Chitosan + Etch-and-rinse adhesive; (C) Self-etch adhesive (D) Chitosan + Self-etch adhesive. R, resin. HL, hybrid layer. D, dentin. T, resin tags

The intra-examiner kappa agreement index was 0.92 for examiner A and 0.90 for examiner B. The inter-examiner kappa (A and B) value was 0.85. SEM characterization of the bonding interfaces for the non-aged and aged groups is presented in Figure 4 and Figure 5. A homogeneous hybrid layer and good adhesive interface were observed in both non-aged and aged groups. Long resin tags were observed most frequently in the etch-and-rinse groups (control and chitosan-treated).

### Fourier-transform infrared spectroscopy

**Figure 6:**
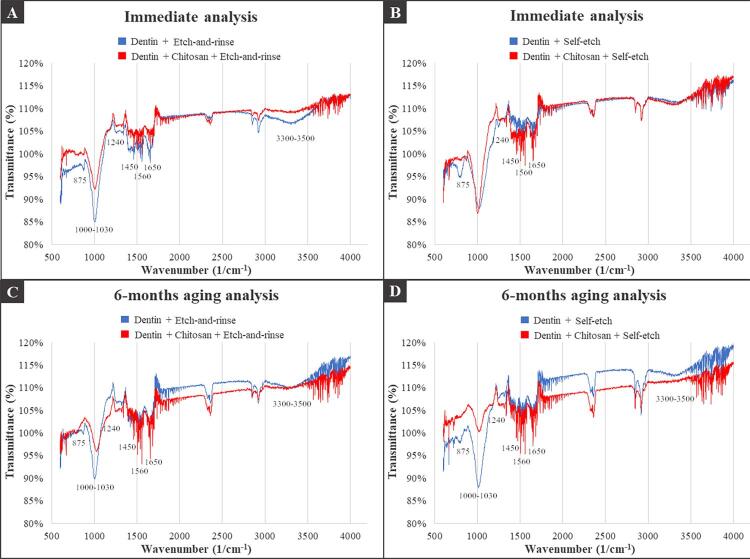
FTIR spectroscopy images of specimens with and without chitosan treatment: A) Etch-and-rinse adhesive - Immediate analysis; B) Self-etch adhesive - Immediate analysis; C) Etch-and-rinse adhesive - 6-months degradation; D) Self-etch adhesive - 6-months degradation. Carbonate (875 cm-1). Phosphate (1000-1030 cm-1). Amide III (1240 cm-1). CH2 (1450 cm-1). Amide II (1560 cm-1). Amide I (1650 cm-1). OH (3300-3500 cm-1)

The organic and inorganic constituents of the dentin included amide I (1650 cm^-1^), amide II (1560 cm^-1^), amide III (1240 cm^-1^), CH_2_ (1450 cm^-1^), phosphate (1000-1030 cm^-1^), carbonate (875 cm^-1^), and OH (3300-3500 cm^-1^). The distribution of the chemical substances remained stable in the immediate analysis and after 6 months of aging in all experimental groups regardless the adhesive system and the type of dentin treatment. A small spacing in the graphic transmittance could be noted between the control and the chitosan groups of aged specimens, suggesting some differences in dentin composition. The spectra showed a decrease in the intensity of phosphate (1100 cm-^1^) and carbonate (872 cm-^1^) bands after modification with chitosan. Figure 6 shows the transmittance dispersion graphs of the experimental groups.

## Discussion

Earlier studies have reported the use of chitosan in Dentistry.^2,14,15,19,26^ The favorable results are due to its properties such as removal of the smear layer,^26^ antimicrobial effect,^14^ and the ability to decrease the activity of metalloproteinases,^2^ thereby stabilizing the resin-dentin bond.^17^

In this study, we applied chitosan before the adhesive protocol to preserve the hybrid layer. The resin-dentin bond strength was assessed, and chemical and morphological analyses of the interface were performed. The µTBS test simulates the forces exerted on the restorative material in the oral cavity.^27^ One of the advantages of this test is that each tooth produces multiple specimens and the tensile force is concentrated on the bonded interface.^28^ The variation coefficient is lower in the µTBS test than in other tensile tests.^29^

No significant difference was observed in the bond strength of the resin-dentin interface between the adhesive systems used in the restorative procedures (etch-and-rinse and self-etch). These results are consistent with earlier investigations that achieved similar bond strength values in dentin for the etch-and-rinse and self-etch adhesive systems.^30^ In contrast, other studies have reported a better dentin-bonding preservation of the self-etch adhesive technique than the etch-and-rinse technique.^31^ This difference in the results was probably due to the differences in methodologies and materials.

The etch-and-rinse technique may result in good resin impregnation into dentin with long tags, allowing mechanical interlocking with the substrate.^4^ Self-etch adhesives can impregnate the underlying dentin by their intrinsic acidity, incorporating the smear layer in the hybrid layer.^7^ Moreover, using the smear layer as a bonding substrate avoids collagen collapse by acid.^8^ According to a meta-analysis on adhesive systems,^32^ there was no difference in the longevity between the etch-and-rinse and self-etch adhesives at 3, 6, and 12 months of aging. Both adhesive systems used in this study are considered gold standards for bond strength studies and, despite using different mechanisms, they provide predictable bond strength to dentin.^30^ Despite that, studies have provided morphological evidences of adhesive elution and/or hydrolytic degradation of collagen matrices after long-term storage, even when using gold standard materials.^33^ So, new strategies have been researched to improve restorations’ bond strength and durability, for example the MMP inhibitors, such as chitosan.

The efficient adhesion can explain the predominance of mixed failures in chitosan-treated specimens restored with a self-etch adhesive. Adhesive failure reflects intense stress distribution within the interface,^28^ and it is the most common failure type found in µTBS tests, as we found in our study.

According to a study on *in vitro* testing of composite bonds to dentin using microtensile tests,^34^ 6-month storage in water can be considered a medium to long aging period. Hence, it was used to simulate *in vivo* degradation conditions.^17^ Deterioration of the resin-dentin interface usually occurs due to enzymes that break down collagen.^35^ To accelerate the collagen degradation process, Clostridium histolyticum collagenase was used in the aged groups.^36^ Although *C. histolyticum* is not directly involved in dental infections, protease from these bacteria has some structural similarity with other bacterial enzymes.^37^

Both control and chitosan-treated interfaces were preserved after degradation. No significant statistical difference was observed between the immediate and 6-month μTBS values. The difference in μTBS values (p=0.004) was found between treated and non-treated groups. Dentin treated with chitosan had better immediate bond strength of resin, which was maintained after degradation. Therefore, chitosan gel can be used to improve immediate adhesion and preserve bond durability. In a previous *in vitro* study,^17^ we verified that chitosan did not influence the collagenolytic activity, but preserved the resin-dentin bonds after 12-month water storage. The structure of chitosan has free amino and hydroxyl groups, besides positive charges that form a cross-linkage with dentin collagen through ionic complexes, producing a mechanically strong fibril chain and raising the mechanical performance of restorations.^2,38^ According to a previous study, the crosslinking within collagen fibril can occur in 12 h,^38^ so we can suppose that the immediate bond strength in our study (24 h) was improved because of this process. Moreover, chitosan can decrease the enzymatic activity of collagenase^2^, since it is a calcium chelator.^39^ These properties improve the bond strength of the composite resin to the dentin surface^38,40^ and are following the results of this study, explaining the improvement in bond strength of chitosan-treated groups compared to the control ones (without chitosan). Thus, the first null hypothesis was rejected.

The sticks were cut after the aging of the adhesive surface to reproduce the real oral situation, in which the outer part of the restorations can be more affected than the inside one. On the other hand, it could be a limitation of this study due to the difficulty for collagenase to reach the whole interface and act differently on each part. Our methodology, most closely to the clinical condition, could explain the contrast with the literature. We found higher bond strength values after aging than the studies that degrade the sticks and not the entire restoration.^22,24^ Another limitation for this laboratorial study was the impossibility of using a balanced-tooth dependency and a complete split-tooth design. We used a random teeth distribution due to difficulty to find four recently extracted caries-free teeth from the same person (from different participants).

SEM and EDS analyses allowed the characterization of the morphology and chemical composition of the specimens.^22^ No significant modification was observed in the chemical composition in EDS. The original relationship between organic and inorganic components was maintained, so the second null hypothesis was accepted. SEM images revealed a uniform hybrid layer and good adhesive interface in all groups, corroborating literature.^4^ Thus, the third null hypothesis was also accepted. The prevalence of long resin tags observed in the etch-and-rinse groups was expected and could be explained by acid etching, which favors resin penetration into dentinal tubules.^4,6^ However, the self-etch system allows resin monomers to penetrate the smear layer into mineralized dentin and form a strong hybrid layer.^7^

The chemical composition was also assessed by the versatile technique FTIR spectroscopy, which characterizes structural materials in the carbon family from the interaction of infrared radiation with substances.^41^ This technique has the advantage of non-destructive and real-time measurement, allowing quantitative and qualitative determination.^41^ This study performed qualitative analysis of organic and inorganic substances in dentin tissues using FTIR transmittance. Transmittance is the capability of infrared radiation to pass through the sample components.^42^ The peak formation displayed in Figure 6 indicates a drop in transmittance, indicating that a fraction of the spectrum (determined by specific wavenumber range) was absorbed by a particular chemical constituent present in the sample. Therefore, the specific wavenumber ranges absorbed by all samples were 1650 cm^-1^, 1560 cm^-1^, 1240 cm^-1^, 1450 cm^-1^ 1000-1030 cm^-1^, 875 cm^-1^, and 3300-3500 cm^-1^, corresponding to amide I, amide II, amide III, CH_2_, phosphate, carbonate, and stretch OH, respectively.^43^

The same substances were observed in the chemical composition of the groups according to the adhesive system or dentin treatment (with or without chitosan). This finding emphasizes that chitosan maintains the stability of the mineral and organic dentin compounds (mainly phosphate and amide groups) both in the immediate and in the aged (6 months) specimens even after dentin etching. In the aged groups, differences in the dentin composition of each tooth can explain the spaces in the visual transmittance between the control specimens and the chitosan specimens.

Chitosan is a biopolymer consisting of B-(1-4)-2-amino-2-deoxy-D-glucopyranose and B-(1-4)-2-acetamide-2-deoxy-D-glucopyranose derived from the chitin deacetylation reaction.^12,13^ The main bands founded in the chitosan spectrum were 1650 cm^-1^ (amide I), 1560 cm^-1^ (amide II), 1700 cm^-1^ and 3450 cm^-1^ (stretch OH),^44^ which correspond to the chemical composition of collagen.^43^ The bands corresponding to the amide group in dentin also correspond to the main bands of the chitosan spectrum,^38^ explaining the strongly overlap on spectra of the adhesive interface. This overlap indicates the presence of chitosan in the sample and its interaction with dentin collagen.^38^ Moreover, the spectra showed a decrease in the intensity of phosphate (1100 cm-^1^) and carbonate (872 cm-^1^) bands after using chitosan, suggesting the interaction and biomodification, since chitosan molecule contains reactive sites in its composition,^13^ which allow chemical substitutions.^45^So, the fourth null hypothesis was rejected.

The outcomes of our study encourage further investigations with novel variations of chitosan, aiming to increase the bonding durability of the adhesive materials to dental substrates and to reveal additional properties of this unique biomaterial.

## Conclusion

According to the results, it is possible conclude that:

Chitosan improved the bond strength of the adhesive interface compared to control without treatment;

Both adhesive systems had the same performance in the bond strength of resin to dentin;

After degradation, the chitosan gel preserved the µTBS of the adhesive interface with higher values than non-treated specimens;

Neither chemical elements nor morphology of the adhesive interface was changed, but IR spectrum suggests biomodification of dentin by chitosan.
